# Reply to Erren et al. Chronodisruption: Origin, Roots, and Developments of an 18-Year-Old Concept. Comment on “Desmet et al. Time-Restricted Feeding in Mice Prevents the Disruption of the Peripheral Circadian Clocks and Its Metabolic Impact during Chronic Jetlag. *Nutrients* 2021, *13*, 3846”

**DOI:** 10.3390/nu14020316

**Published:** 2022-01-13

**Authors:** Louis Desmet, Theo Thijs, Rosalie Mas, Kristin Verbeke, Inge Depoortere

**Affiliations:** 1 Translational Research Center for Gastrointestinal Disorders, Gut Peptide Research Lab, University of Leuven, Gasthuisberg, 3000 Leuven, Belgium; Louis.desmet@kuleuven.be (L.D.); Theo.thijs@kuleuven.be (T.T.); r.mas@outlook.be (R.M.); 2 Translational Research Center for Gastrointestinal Disorders, Laboratory of Digestion and Absorption, University of Leuven, Gasthuisberg, 3000 Leuven, Belgium; Kristin.verbeke@kuleuven.be

We would like to thank Erren et al. for their interest in and comments on our recent paper “Time-Restricted Feeding in Mice Prevents the Disruption of the Peripheral Circadian Clocks and Its Metabolic Impact during Chronic Jetlag” [1].

Erren et al. have commented that we provided a literature reference for the definition of chronodisruptors, but not for the definition of chronodisruption itself [2]. We agree with the authors that it is useful to add a reference concerning the origin of the definition of chronodisruption [3] in order to help scientists to learn about, challenge, falsify, or expand the increasingly used concept of chronodisruption.
